# LC-MS/MS-based shotgun proteomics identified the targets of arthritis-related microRNA

**DOI:** 10.1186/ar3637

**Published:** 2012-02-09

**Authors:** Riho Kurata, Tomo Yonezawa, Hideki Nakajima, Shyuji Takada, Hiroshi Asahara

**Affiliations:** 1 Department of Systems BioMedicine, National Research Institute for Child Health and Development, Setagaya-ku, Tokyo 157-8535, Japan; 2 Department of Molecular Life Sciences, Basic Medical Science and Molecular Medicine, Tokai University School of Medicine, Isehara, Kanagawa, Japan; 3 Department of Pediatric Hematology and Oncology Research, National Research Institute for Child Health and Development, Setagaya-ku, Tokyo 157-8535, Japan; 4 Department of Systems BioMedicine, Tokyo Medical and Dental University, Bunkyo-ku, Tokyo 113-8510, Japan; 5 Core Research for Evolutional Science and Technology, Japan Science and Technology Corporation, Saitama 332-0012, Japan

## 

microRNAs (miRNAs), which are class of post-transcriptional regulators such as short 19 to 23-nucleotide non-coding RNAs, complementarily bind seed sequences in the 3'-untranslational region of multiple target mRNAs, resulting in their suppression of translation or degradation [1]. In the former case, since the mRNA expression of the targets does not any change, transcriptomics approach, such as expression array, cannot identify the targets.

Recent studies shed light on the fine-tuning mechanism of miRNAs in myriad biological processes including development [2], tumorigenesis [3] and inflammation [4]. We have identified enhancement of mir-146a expression in rheumatoid arthritis synoviocyte and macrophages [5], whilst suppression of them in osteoarthritis [6]. Another group also have identified the enhancement of mir-146a and mir-155 in response to bacterial pathogen such as lipopolysaccaride [7]. Recently, mice lacking of mir-155 are resistant to collagen-induced arthritis (CIA) [8], whilst administration of mir-146a complexed with aterocollagen into joint attenuates pathological condition of CIA [9]. These results indicate that mir-146a and mir-155 plays an important role for developing arthritis and inflammation. However, the targets of both two miRNAs and their molecular mechanisms are not still fully identified.

In this study, in order to identify the targets of them in translational level, we established gain of function models using adenovirus- and CMV promoter-mediated overexpression in several culture models and performed liquid chromatography-tandem mass spectrometry-based shotgun proteomics in these models.
